# Lifetime risks and health impacts of hemorrhagic and ischemic stroke in South Korea

**DOI:** 10.1038/s41598-020-71439-3

**Published:** 2020-09-03

**Authors:** Soyeon Cheon, Hyangsook Lee, Jiyoon Won, Bo-Hyoung Jang, Jung-Der Wang

**Affiliations:** 1 Department of Public Health, National Cheng Kung University College of Medicine, No. 1, University Road, Tainan, Taiwan; 2 Acupuncture and Meridian Science Research Center, College of Korean Medicine, Kyung Hee University, Seoul, Korea; 3 Department of Science in Korean Medicine, Graduate School, Kyung Hee University, Seoul, Korea; 4 Department of Preventive Medicine, College of Korean Medicine, Kyung Hee University, Seoul, Korea

**Keywords:** Epidemiology, Outcomes research, Cardiovascular diseases, Public health

## Abstract

This study is aimed toward estimating the lifetime risks, life expectancy, expected years of life lost (EYLL), and lifetime costs related to different subtypes of stroke in South Korea. We included 13,994 patients diagnosed with stroke (ICD-10, I60-I63) in the National Health Insurance Service-National Sample Cohort of Korea between 2006 and 2015. Lifetime risks were calculated using the cumulative incidence rate for patients aged 18–84. Lifetime survival data were obtained through the Kaplan–Meier method and extrapolated with a rolling-over extrapolation algorithm. The lifetime costs were estimated by multiplying the average monthly expenditures with the survival probabilities and adding the values over lifetime. The lifetime risks of stroke in Korea have been decreasing consistently over the last decade with the exception of subarachnoid hemorrhage in females, which appears to have slightly increased. The EYLL is higher in hemorrhagic stroke than in ischemic stroke (6–9.7 vs. 4.7). Expected lifetime costs reimbursed by the NHIS would amount to about $71,406 accompanied with $14,921 copayment from the patients for hemorrhagic stroke, and $50,551 and $11,666, respectively, for ischemic stroke. Further studies are warranted to combine survival with quality of life and functional disability to obtain a more detailed outcome assessment of the potential impact of the prevention of stroke.

## Introduction

Being the second most common cause of deaths and the third most common cause of disability worldwide, strokes result not only in mortality but also in morbidities affecting the lives of survivors and their family members^1^. Among the top 30 causes of years of life lost, only seven of them also appear in the top 30 causes of years lived with disability, including stroke^2^. With advances in treatment, as well as the control of hypertension for prevention of stroke during the last couple of decades, we are witnessing a decline in stroke incidence and mortality^3^. In a recently updated report of stroke statistics in South Korea, the age- and sex-standardised incidence rates of first-ever stroke showed a gradual decrease from 105.8 per 100,000 person-years in 2007 to 92.2 in 2013^4^. However, an ageing population as well as population growth itself still continues to increase the absolute burden of stroke^5^, which is also the case in Korea. Stroke has recently dropped to the third most common cause of deaths after cancer (all types combined) and heart disease^6^, but the prevalence and medical expenses related to stroke have been continuously growing over the last decade^7^.

An increasing burden of stroke is inevitable unless there is a significant drop in the incidence rate, and therefore, along with improving quality of care, policy makers must emphasise effective prevention of stroke. Especially in countries with an ageing and/or aged population, there has been a continuous emphasis placed on preventive medicine^1,8^. Nonetheless, the difficulties in quantifying the anticipated savings of life years and/or costs due to prevention have been an obstacle for preventive medicine in terms of receiving sufficient resources^9–11^. In addition to determining the incidence rate as the first step to show the impact of prevention, it is also necessary to quantify the consequences (or pay-off in economic terms) of stroke in units that are directly comparable to other clinical services in order for policy makers to allocate resources fairly.

Although there are a number of studies that describe the various outcomes of stroke in Korea^12–15^, to our knowledge, there is no evidence of the lifetime health benefits and costs savings resulting from successful prevention of stroke. A newly developed method^16–18^ for extrapolating lifetime outcomes beyond a given observation period, which has been approved by international scholars^19^, now makes it possible to obtain an accurate estimation of life expectancy (LE), expected years of life lost (EYLL), and lifetime healthcare expenditures for various diseases. By applying this novel method to real-world Korean data, we aimed to estimate: (1) the lifetime risks, (2) LE and EYLL, and (3) direct medical costs over lifetime for insurers and patients by utilizing a decade of national health insurance reimbursement data from patients diagnosed with hemorrhagic and ischemic stroke in South Korea.

## Results

**Figure 1:**
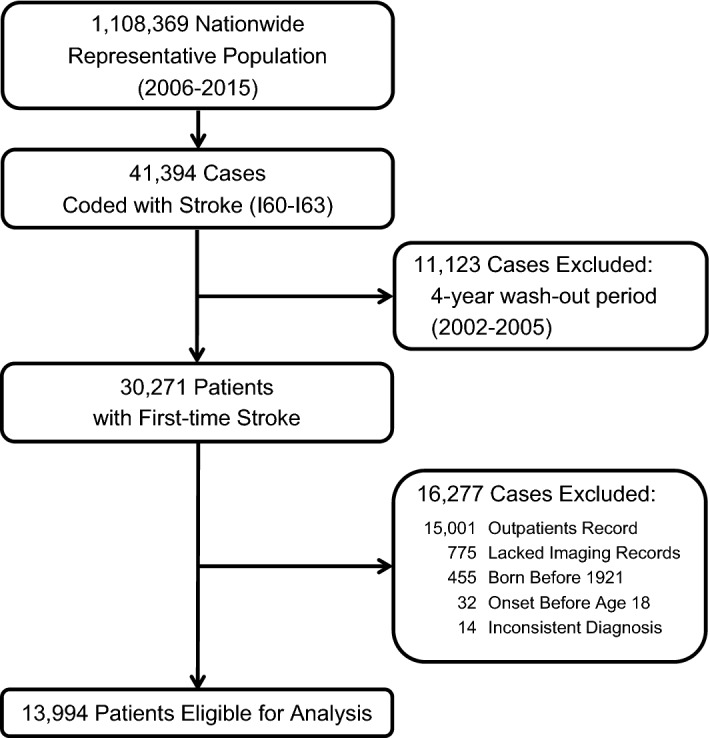
Flow diagram for establishing the index cohort.

Reimbursement records for 41,394 patients coded with hemorrhagic or ischemic stroke during the period between 2006 and 2015 were found, and among them, 30,271 patients had a first-time diagnosis of stroke (Fig. 1). To assure the validity of diagnosis, we excluded 15,776 cases without any hospitalization records and/or imaging evidence of either computed tomography (CT) or magnetic resonance imaging (MRI). Furthermore, 487 patients who did not have accurate age information or who were younger than 18 years old, and 14 patients without consistent diagnosis on the index date were excluded. A total of 13,994 patients were eligible for analysis out of the 1 million sample population cohort. Among the patients who were excluded due to a lack of hospitalization records (15,001), 5,566 patients had made at least three visits to an outpatient clinic.

Demographic and clinical characteristics at diagnosis for the included and excluded patients are summarised according to different subtypes of stroke in Table 1 and Supplementary Table S4. Approximately 73.5% of the patients had a cerebral infarction (CI), followed by 13.4% with intracerebral hemorrhage (ICH), and 8.4% with subarachnoid hemorrhage (SAH), which corroborates with a recent report^20^, indicating the validity of the included cohort. A post-hoc analysis showed that there were statistically significant differences among the age distributions of patients in the different stroke subtypes (Kruskal–Wallis test, P < 0.0001), where patients with SAH were the youngest. About 10% of the patients in the cohort were beneficiaries of medical aid services in Korea, which is higher than the average 3.4% of beneficiaries in the total population reported between 2006 and 2015^21^. The most common comorbidity among the stroke patients was hypertension, followed by hyperlipidemia and diabetes. When compared with the included patients, the excluded patients generally showed lower prevalence rates of pre-existing comorbidities, which is consistent with our hypothesis that these are, at most, relatively mild cases of stroke^22^. Patients with ischemic stroke scored the lowest on the stroke severity index (SSI), indicating less severe clinical conditions during their first hospital stay due to stroke compared with those with hemorrhagic stroke (median 4.1 vs. 12.1, respectively).

**Table 1:** Baseline demographic and clinical characteristics of stroke patients from the Korean NHIS-National Sample Cohort.

	SAH N = 1,182	ICH N = 2,022	Other NIH N = 508	CI N = 10,282	Overall N = 13,994	Excluded^a^ N = 5,566
**Male**	458 (38.8)	1,152 (57.0)	344 (67.7)	5,584 (54.3)	7,538 (53.9)	2,492 (44.8)
**Age, years (mean)**	56.3	61.6	64.9	67.7	65.7	66.1
18–44	229 (19.4)	221 (10.9)	53 (10.4)	431 (4.2)	934 (6.7)	244 (4.4)
45–54	343 (29.0)	446 (22.1)	75 (14.8)	1,255 (12.2)	2,119 (15.1)	668 (12.0)
55–64	285 (24.1)	474 (23.4)	81 (15.9)	2,028 (19.7)	2,868 (20.5)	1,281 (23.0)
65–74	190 (16.1)	453 (22.4)	150 (29.5)	3,151 (30.7)	3,944 (28.2)	1,976 (35.5)
75–84	111 (9.4)	362 (17.9)	121 (23.8)	2,863 (27.8)	3,457 (24.7)	1,257 (22.6)
85 +	24 (2.0)	66 (3.3)	28 (5.5)	554 (5.4)	672 (4.8)	140 (2.5)
**Residence area**						
Seoul metropolitan city	215 (18.2)	356 (17.6)	113 (22.2)	1,562 (15.2)	2,246 (16.1)	1,115 (20.0)
Other metropolitan cities	325 (27.5)	515 (25.5)	95 (18.7)	2,428 (23.6)	3,363 (24.0)	1,191 (21.4)
Non-metropolitan area	642 (54.3)	1,151 (56.9)	300 (59.1)	6,292 (61.2)	8,385 (59.9)	3,260 (58.6)
**Insurance type**						
Self-employed insured	441 (37.3)	777 (38.4)	166 (32.7)	3,407 (33.1)	4,791 (34.2)	1,767 (31.8)
Employed insured	666 (56.4)	1,073 (53.1)	284 (55.9)	5,736 (55.8)	7,759 (55.5)	3,114 (56.0)
Medical aid beneficiary	75 (6.4)	172 (8.5)	58 (11.4)	1,139 (11.1)	1,444 (10.3)	685 (12.3)
**Comorbidities**						
Atrial fibrillation	41 (3.5)	90 (4.5)	40 (7.9)	1,130 (11.0)	1,301 (9.3)	127 (2.3)
CHF	36 (3.1)	76 (3.8)	29 (5.7)	697 (6.8)	838 (6.0)	125 (2.3)
CKD	11 (0.9)	75 (3.7)	16 (3.2)	232 (2.3)	334 (2.4)	65 (1.2)
COPD	75 (6.4)	167 (8.3)	64 (12.6)	1,076 (10.5)	1,382 (9.9)	303 (5.4)
Diabetes	255 (21.6)	614 (30.4)	154 (30.3)	4,025 (39.2)	5,048 (36.1)	909 (16.3)
Hyperlipidemia	251 (21.2)	563 (27.8)	149 (29.3)	5,774 (56.2)	6,737 (48.1)	936 (16.8)
Hypertension	595 (50.3)	1,419 (70.2)	298 (58.7)	6,669 (64.9)	8,981 (64.2)	1509 (27.1)
IHD	142 (12.0)	279 (13.8)	92 (18.1)	1,986 (19.3)	2,499 (17.9)	563 (10.1)
TIA	167 (14.1)	61 (3.0)	33 (6.5)	679 (6.6)	940 (6.7)	286 (5.1)
**SSI (median)**	15.6	12.1	9.7	4.1	5.8	NA

Values are numbers (percentage) unless stated otherwise.

*CHF* congestive heart failure, *CI* cerebral infarction, *CKD* chronic kidney disease, *COPD* chronic obstructive pulmonary disease, *ICH* intracerebral hemorrhage, *IHD* ischemic heart disease (including myocardial infarction), *NA* not applicable, *NHIS* National Health Insurance Service, *NIH* non-traumatic intracranial hemorrhage, *SAH* subarachnoid hemorrhage, *SSI* stroke severity index (ranging from 4.1 to 27.11; a higher score indicates more severe status), *TIA* transient ischemic attack.

^a^This number is patients who have visited an outpatient clinic more than three times with a stroke diagnosis code.

### Lifetime risks of stroke

**Figure 2:**
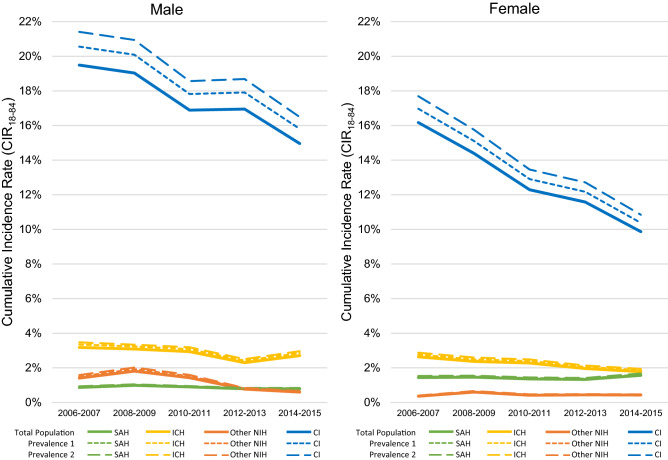
Lifetime probability of developing different subtypes of stroke. CIR_18–84_ of different subtypes of stroke from 2006 to 2015. The three line patterns represent different CIR estimations with slightly different denominators: the solid line indicates the number of total population from vital statistics in each strata; the dotted and broken lines indicate estimations after subtracting the number of prevalent cases of stroke from the denominator based on two different prevalence analyses from Korea (prevalence 1 and 2)^4^. *CI* cerebral infarction, *ICH* intracerebral hemorrhage, *NIH* non-traumatic intracranial hemorrhage, *SAH* subarachnoid hemorrhage.

The lifetime risks of hemorrhagic and ischemic strokes, calculated using the cumulative incidence rate (CIR), in males and females are summarised in Fig. 2 and Supplementary Table S5. In general, the lifetime risk of stroke for males was higher than females, except for SAH, during the last decade. The estimated lifetime risk of ischemic stroke (CIR_18–84_) seemed to consistently decrease over the 10-year observation period, from 19.5 to 15% and from 16.1 to 9.9%, for males and females, respectively. When adjusted with prevalence rates, the estimated results were slightly higher than the original estimates, due to the smaller size of the denominator. The lifetime risk of hemorrhagic stroke showed a negligible but decreasing trend over the years, except for SAH in females.

### Validation of the extrapolation method

Mean survival for the 10-year period was estimated using the Kaplan–Meier method and the semi-parametric extrapolation method for comparison, for which the results are summarised in Supplementary Table S6. The extrapolated estimates based on the first 5 years of follow up were close to those obtained using the Kaplan–Meier method, showing relative biases of less than 1.4% in all subtypes of stroke, thus supporting the validity of this method.

### Estimation of life expectancy (LE) and expected years of life lost (EYLL)

The average life span of the age-, sex-, and calendar year-matched reference population was similar among the different types of hemorrhagic and ischemic stroke (83.2–85.1 years), but the estimated LE after diagnosis and EYLL for the stroke cohort varied among the different subtypes (Table 2, Supplementary Fig. S1). Patients with SAH were estimated to have the highest LE (17.9 years) yet also the highest EYLL (9.7 years), which was followed by patients with ICH, with 7.8 years of EYLL.

**Table 2:** Life expectancy (LE), expected years of life lost (EYLL), and lifetime direct medical costs per case by different subtypes of stroke in Korea.

Stroke type	N	Age at Dx	LE after Dx	EYLL	Expenditures covered by NHIS	Copayment expenses
SAH	1,182	56.31 (0.40)	17.90 (1.76)	9.66 (1.76)	58,741 (4,031)	12,290 (1,052)
ICH	2022	61.60 (0.31)	14.44 (0.96)	7.80 (1.00)	71,406 (4,328)	14,921 (867)
Other NIH	508	64.94 (0.65)	13.30 (2.05)	6.01 (2.19)	49,911 (5,526)	9,684 (1,037)
CI	10,282	67.65 (0.12)	12.75 (0.50)	4.68 (0.49)	50,551 (1,716)	11,666 (344)

US$1 = 1,131.2 KRW in 2015.

Values are mean (SE) in years or US$.

The medical costs are adjusted with 3% annual discount rates here.

*CI* cerebral infarction, *Dx* diagnosis, *ICH* intracerebral hemorrhage, *N* number, *NHIS* National Health Insurance Service, *NIH* non-traumatic intracranial hemorrhage, *SAH* subarachnoid hemorrhage, *SE* standard error.

### Lifetime healthcare expenditures

The lifetime healthcare expenditures for hemorrhagic and ischemic strokes for the Korean National Health Insurance Service (NHIS) and the copayment expenses for patients are summarised in Table 2 and Supplementary Table S7. Based in the calculations, ICH resulted in the largest financial burden to the NHIS, with an estimated $71,406, which was followed by SAH ($58,741), and CI ($50,551) for each incident stroke case when adjusted with a 3% annual discount rate. Copayment expenses comprised approximately 16.2–17.3% of the total costs for hemorrhagic stroke, whereas the proportion was 18.8% for ischemic stroke.

## Discussion

The present study is the first to quantify both the likelihood (Fig. 2) and consequences (Table 2) of hemorrhagic and ischemic strokes in Korea, which will provide stakeholders with an opportunity to compare the potential impacts of prevention versus other healthcare services. We found that the lifetime risks of ischemic stroke (CIR_18–84_) have been decreasing for both genders over the last decade, while those for different subtypes of hemorrhagic stroke did not show such an obvious trend, especially among males with ICH and females with SAH (Fig. 2). Although the lifetime risk is much higher in ischemic stroke than in the different subtypes of hemorrhagic strokes, the EYLLs and lifetime health expenditures are higher in the latter events. Namely, the EYLL (or, loss of life expectancy) for patients with hemorrhagic strokes would be 6–9.7 years with lifetime healthcare expenditures of $59,595–$86,327, while those of ischemic stroke would be 4.7 years and $62,217 per patient (Table 2).

Before making any more inferences, we must first justify the validity of these estimations. First, our results of lifetime risks or CIR_18–84_ corroborate those of another study of stroke in Korea^3^. Since the lifetime risk of a disease has an intuitive interpretation of personal risk under probability terms and can be directly compared across different time periods^23,24^ and countries^25–29^, this information could quickly catch the attention of both lay people and policy makers. Second, since accurate quantifications of LE and lifetime healthcare expenditures are crucial for a fair comparison of the societal impacts between prevention and all other direct clinical services, we must also validate our extrapolation method used to determine lifetime survival. In addition to the theoretical justifications^18,19^, we performed empirical validations by using the first five years of data to extrapolate to the end of the 10th year and compared them with actual Kaplan–Meier’s estimates made for the 10 years of follow-up. Because the relative biases were all below 1.4% (Supplementary Table S6), this assured the accuracy of the estimated lifetime survival function based on the 10 years of follow-up from 2006 to 2015, which was much closer to the LE of usual strokes (Table 2). Third, we estimated EYLL as the indicator of potential savings from prevention of loss of LE. Because EYLL is the difference between the LE of the stroke cohort and that of the age-, sex-, and calendar year-matched referents, it quantifies the lifetime health benefits brought about by successful prevention of a case of stroke. In addition, because it has already been adjusted for differences in age and sex^30,31^, it inherently controls lead time bias when early diagnosis is applied as a strategy for prevention. Matching for the same calendar year would adjust for progressive improvements in medical technology over time. Therefore, these results are presented in units that make prevention directly comparable for value with all other direct clinical services such as diagnosis, treatment, rehabilitation, alternative medicines, etc., and/or other health conditions, and thus could aid health policy makers in their decision-making processes related to more equitable and efficient allocation of scarce resources.

Exploration of trends of lifetime risks of stroke subtypes raises issues of prevention strategies in Korea: the lifetime risk of ischemic stroke (CIR_18–84_) dropped to about 1 in 6 males and 1 in 10 females from 2014 to 2015. The lifetime risks for hemorrhagic stroke also showed a small yet decreasing trend (Fig. 2). While other types of stroke showed higher lifetime risk in men than in women, consistently over the last decade, this appeared to be reversed in the case of SAH (e.g., in the time period between 2014 and 2015, for males, 1 in 127 and for women, 1 in 64). Other studies have reported similar lifetime risk of stroke between men and women^3,26^, and the same trend of a much greater lifetime risk of SAH in women was observed in a study conducted in Japan^26^. Previous studies have reported that men have a higher stroke incidence than women in general, but in the case of SAH, the incidence is higher in women than in men^32,33^. The mechanism behind this phenomenon is still unclear, but some studies propose hormonal factors as a possible explanation^33–35^. The estimates did not differ to any great degree when the population at risk for calculation was adjusted by two different reported prevalence rates of stroke in Korea^4^. In these prevalence rates, new cases from the same year were also included, so the estimates from this sensitivity analysis would lead to a minor over-estimation, or the upper bound of the CIR.

Our estimation of lifetime expenditures is based on incident cases, and as such is more accurate compared with previous studies based on prevalent cases^13,36–38^. While such studies reported a comprehensive approach, including both direct and indirect costs, many components of the costs were calculated by simply multiplying specific pre-determined amounts by the number of stroke patients. On the other hand, the strength of the current study lies in the fact that the analysis is from real-world data that incorporates all logged expenses in the NHIS reimbursement dataset after the diagnosis of stroke. Another recent study has estimated the 5-year cumulative costs of 3,393 patients of ischemic stroke in Korea^39^. Although they also analyzed the NHIS dataset, unlike our national sample cohort, their customized dataset allowed their estimates to include costs of long term care. However, their 5-year cumulative costs were summed up throughout the 5 years by taking the average daily costs for those followed for 5 years and were not weighted for survival rates at each time point. In contrast, we applied Kaplan–Meier method to account for survival rate and censored patients at each time points, which would be the average lifetime medical costs for a stroke patient. Furthermore, the rolling-over extrapolation algorithm used in this study has been demonstrated to show accurate, robust results regardless of whether the index cohort’s excess hazard is constant, decreasing, or increasing after censoring observations^18^. Therefore, while previous studies have shown a rising burden of stroke year after year, the lifetime health expenditures of different subtypes of stroke estimated in the present study provide additional evidence on how much financial burden per case could be avoided for the Korean NHIS and individuals thanks to the prevention of stroke.

There are several limitations that must be acknowledged in this study. First, while the NHIS dataset has many advantages, such as being real-world data representative of Korea, large in size, with a 10-year follow up, and relatively free of recall bias, it involves input of data (e.g., diagnosis) from various levels of hospitals and clinics, so the validity of the diagnoses of different subtypes of stroke may thus be in question. However, this concern could be partially dealt with by having clear inclusion and exclusion criteria. In this study, we included stroke patients who were hospitalised with diagnosis codes I60–I63 (ICD-10) accompanied with imaging records such as CTs and MRIs, to enhance the validity of the diagnosis. While this step was crucial since the inclusion of only cases with a valid diagnosis was a prerequisite for the entire study, it may have resulted in excluding some mild stroke cases that did not require hospitalization. The potential impact of this inclusion criteria thus could have resulted in underestimation of the results. Second, the estimates were based on currently available data from 2006 to 2015, but the true outcomes for these patients in the future may reflect improvements in stroke care. This means that the lifetime survival results from our study could easily have been underestimated, while the EYLL could have been over-estimated. Therefore, the results should be interpreted carefully with consideration of the possible direction of such effects. Another limitation of this study is that we only analyzed the lifetime cost of stroke in terms of direct medical costs that are covered by the NHIS. In Korea, however, NHIS reimburses over 80% of the care provided to patients with stroke in hospitals, meaning that our study did include most of the expenses. Therefore, the missing portion would only lead to an underestimation of the total direct medical costs^13^. Also, many studies on the cost of illness have laid out different types of cost, including direct non-medical costs as well as indirect costs such as productivity loss. Although we did not include other compositions of costs here, we could refer to previous reports to get an overall idea of the total costs. For example, a review on the indirect costs of stroke have reported the median proportion of indirect costs to total costs to be 32%^40^; thus, with our results for direct costs, one may roughly estimate the total lifetime costs. For a more in-depth understanding, further studies on different types of costs, such as indirect costs from productivity loss, are warranted.

Future studies may include further stratification of the cohort, such as different levels of stroke severity, different types of comorbidities, or different treatment modalities. Especially in Korea, where many people seek traditional Korean medicine for stroke care^41^, it would be worthwhile to establish a cohort of patients who utilised both conventional and traditional Korean medicine to see the outcome or consequences of integrated care. Also, other outcomes reflecting important values in healthcare, such as quality of life or years living with functional disability could be integrated with the results from the current study to provide further evidence on lifetime outcomes of stroke in Korea.

## Conclusion

For prevention technology to be fairly compared with other healthcare services in order to determine resource allocations, strong evidence that comprises both likelihood and consequences of events is necessary. While the lifetime incidence of stroke appears to have decreased in the last decade, except for SAH in females, the health and financial impact of stroke across the lifetime horizon is still significant, and this burden could be alleviated by successful prevention. The results of this study, together with future works on other important outcomes, such as quality of life and functional disability, may provide the best available real-world evidence for economic evaluation of outcomes of all healthcare services, including prevention of stroke in Korea.

## Methods

### Data source

We used the Korean National Health Insurance Service-National Sample Cohort (NHIS-NSC) from January 2002 to December 2015 to construct our study cohort of adult patients suffering a first-time stroke in South Korea. Access to the database can be obtained by submitting an application to the Korean National Health Insurance Service (NHIS) with ethics approval from the researcher’s institutional review board and a study proposal. The NHIS–NSC is a representative population-based cohort established in 2002. It includes various information, such as participants’ insurance eligibility, diagnostic work-ups and treatments in both clinics and hospitals, and claimed reimbursement data^42^. More details on how the dataset was constructed and maintained can be found in other publications^42,43^. This study was approved by the institutional review board of Kyung Hee University (KHSIRB-18-026), and the board granted a waiver to conduct the study without written informed consent due to its retrospective study design using an anonymized dataset. All methods were performed in accordance with the relevant guidelines and regulations.

### Study population

Adult patients aged ≥ 18 with primary diagnosis codes indicating either hemorrhagic or ischemic stroke from 2006 to 2015 were included in this study. We also searched through records between 2002 and 2005 to look for any prevalent cases or sequelae of stroke. Any individual with such a record was excluded to ensure that all included cases were first-time stroke. Following the diagnostic codes of the ICD-10 (International classification of diseases, 10th revision), codes I60–I62 were used to identify hemorrhagic stroke; I63 was used for ischemic stroke, and I60–I69 was applied to any type of stroke or sequelae of stroke. Because claim data could include exaggerated diagnoses of more severe diseases to avoid rejection, we adopted a common strategy of including only hospitalized cases with stroke to assure the validity of the diagnostic codes^44,45^. In the Korean dataset specifically, the Health Insurance Review and Assessment Service (HIRA) of Korea reported that diagnostic codes on actual hospital inpatient records and the claims dataset were matched 82% overall (94.9% for stroke), but the rate dropped to only 44.4% in outpatient records^46^. Based on this, we are concerned that more than half of those excluded may not have been actual stroke cases, or, the severity of stroke was so mild that they did not require hospitalization, such as a transient ischemic attack. Thus, to assure the validity of the stroke diagnosis in the cohort, patients without hospital admission records were excluded. Among those excluded by this criteria, we identified patients who visited an outpatient clinic at least three times for a comparison with included patients. Patients without diagnostic brain imaging records, i.e., computed tomography (CT) or magnetic resonance imaging (MRI), accurate age information, i.e., patients born before 1921, or without consistent diagnosis on the index date were also excluded.

From the established cohort, we extracted various information, including demographic data, area of residence, insurance type (e.g., self-employed, employed, or medical aid beneficiary), survival status, dates and payments for healthcare utilization, and diagnoses. As baseline clinical characteristics, information on nine comorbidities (atrial fibrillation, congestive heart failure, chronic kidney disease, chronic obstructive pulmonary disease, diabetes, hyperlipidemia, hypertension, ischemic heart disease and transient ischemic attack) that were present before the occurrence of stroke was collected. In addition, we calculated the stroke severity index (SSI)^47^ using information on healthcare utilization during the patients’ first hospital stay. SSI scores range from 4.1 to 27.11, where a higher score means a more severe status. The codes used to define imaging records, comorbidities and predictors of SSI are summarised in Supplementary Tables S1, S2 and S3.

### Lifetime risk of stroke estimated by CIR

From the study population, we identified new age- and sex- specific stroke cases from 2006 to 2015. Then, the number of new cases for each year was divided by the sampling fraction of the corresponding years. This numerator was used to calculate the age- and sex- specific incidence rates for every two years, with the corresponding population at risk from census records^48^ as the denominators. Afterwards, the cumulative incidence rate (CIR) for ages 18–84 (CIR_18–84_) was calculated using the formula below^49^:

$${CIR}_{18-84}=1-exp\left[-{\sum }_{i}\left({IR}_{i}\right)\left({\omega }_{i}\right)\right],\mathrm{ where }\ i=18-\mathrm{24,25}-\mathrm{29,30}-34,\cdots ,80-84$$

In the formula, the incidence rate for the *i*th age group is represented by ${IR}_{i}$, and ${\omega }_{i}$ is the width of the *i*th age group. While the CIR formula provides each year of age an equal weight during calculation, one may be concerned that the relatively large variation, resulting from small sample sizes of denominators and numerators for people living longer than life expectancy, would be over-represented in the final estimation. Therefore, we decided to calculate the CIR of stroke patients for ages 18–84 only.

As a sensitivity analysis, we also estimated the CIR_18–84_ after subtracting the number of prevalent cases from the denominators in each strata based on two different analysis results from Korea^4^.

### Survival analysis and extrapolation

We applied a semi-parametric method that could be used to quantify the lifetime survival function of the index cohort using that of the age-, sex-, and calendar year-matched reference population. This survival function of the reference population was generated from the complete life tables of South Korea^50^ using Monte-Carlo methods^17,18,51^. In brief, the lifetime survival function of the matched referents would have been the LE of the patients if the patients had not developed stroke. For the index cohort, we first used the Kaplan–Meier method to estimate survival from the onset of stroke to the end of follow-up. The survival of the index cohort was extrapolated beyond the end of follow-up to lifetime by establishing a logit transformation of the survival ratio between the index cohort and referents, followed by a rolling-over extrapolation algorithm^18^. Based on the estimated survival function, the LE and EYLL were estimated for four subtypes of stroke, including subarachnoid hemorrhage (SAH), intracerebral hemorrhage (ICH), other non-traumatic intracranial hemorrhage (NIH), and cerebral infarction (CI). In addition, we validated the extrapolation method by adopting the data from 2006 to 2010 and extrapolating to the end of 2015 and then compared them with the actual Kaplan–Meier’s estimate of each subcohort. The relative bias was the difference between the extrapolated estimate and the 10-year follow-up estimate divided by the 10-year follow-up estimate. To obtain the Kaplan–Meier’s estimate, we used Proc Lifetest restricted to the largest observation on the SAS Enterprise Guide (ver. 7.13). The rest of the analyses were conducted in R 3.4.1 using the iSQoL2 (ver. 4.1) package^52^.

### Lifetime healthcare expenditures

Based on the healthcare service utilization fees claimed in our dataset, the lifetime healthcare expenditures covered by NHIS and copayment expenses for patients were calculated in this study. This included direct medical costs incurred for all types of hospitalization, clinical visits, and pharmacies registered in the Korean NHIS from the diagnosis of stroke until being censored or declared deceased. First, we calculated the observed monthly average cost for every case after diagnosis. The cost beyond follow-up was extrapolated using a rolling-over extrapolation algorithm, assuming that medical expenditures typically rise near the end of life. Then, the product of the monthly survival rates and monthly mean costs were summed up to estimate the lifetime cost^18^. Lastly, we adjusted the extrapolated costs with a 3% annual discount rate, and added 0% and 5% rates for the sensitivity analysis. More detailed methods and mathematical proofs have been described in previous reports^17,18^. This calculation was also performed using the iSQoL2 package. The original values coded with Korean Won (KRW) were all converted to US$ based on the average exchange rate in 2015 (1 US$ for 1,131.2 KRW)^53^.

## Supplementary information

Supplementary information

## Data Availability

The datasets generated and analyzed during the current study are not publicly available due to restrictions by the Korean NHIS. However, interested parties may submit a separate application to the NHIS for access. The NHIS accepts applications via their website (https://nhiss.nhis.or.kr), and ethics approval from the researcher’s institutional review board and a study proposal are required^43^. The iSQoL2 package for Windows is freely available at http://sites.stat.sinica.edu.tw/isqol/ and provided with an English manual.
