# Supplementary material for: Lifetime risks and health impacts of hemorrhagic and ischemic stroke in South Korea
Source: Sci Rep. 2020 Sep 3;10:14544. doi: 10.1038/s41598-020-71439-3 (PMC7471302; doi:10.1038/s41598-020-71439-3)
Supplement: Supplementary file 1 — Supplementary information [file 41598_2020_71439_MOESM1_ESM.docx]

Supplementary Materials for Scientific Reports

# Title page

**Lifetime risks and health impacts of hemorrhagic and ischemic stroke in South Korea**

**Soyeon Cheon, KMD, MS^1^; Hyangsook Lee, KMD, PhD^2,3^; Jiyoon Won, KMD, MS^2,3^; Bo-Hyoung Jang, KMD, PhD^4^; Jung-Der Wang, MD, ScD^1^**

^1^ Department of Public Health, National Cheng Kung University College of Medicine, Tainan, Taiwan

^2^ Acupuncture and Meridian Science Research Center, College of Korean Medicine, Kyung Hee University, Seoul, Korea

^3^ Department of Science in Korean Medicine, Graduate School, Kyung Hee University, Seoul, Korea

^4^ Department of Preventive Medicine, College of Korean Medicine, Kyung Hee University, Seoul, Korea

*Correspondence to: Jung-Der Wang

Department of Public Health, College of Medicine, National Cheng Kung University, No.1, University Road, Tainan, Taiwan

Phone: +886-6-2353535 ext. 5600

Fax: +886-6-2359033

E-mail: [jdwang121@gmail.com](mailto:jdwang121@gmail.com)

#
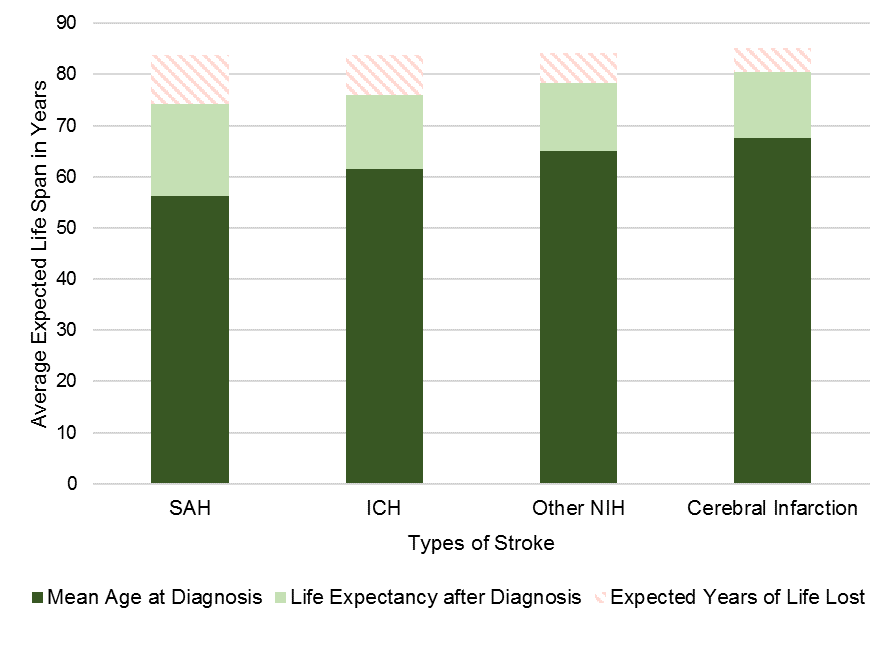
Supplementary Figure

**Supplementary Figure 1.** Average expected life span by different subtypes of stroke. ICH: intracerebral hemorrhage; NIH: non-traumatic intracranial hemorrhage; SAH: subarachnoid hemorrhage.

# Supplementary Tables

Supplementary Table 1. Class codes used to define the imaging records in the study cohort.

| Class | Codes |
| --- | --- |
| Imaging diagnosis | 10 |
| Computed Tomography | C (2006-2007) |
| Magnetic Resonance Imaging | M (2006-2007) |
| Special equipment^*^ | S |

^*^S code was newly implemented in June 2006 to combine C and M codes along with Positron-emission tomography. After 2007, C and M codes were completely replaced by S code.

Supplementary Table 2. ICD-10 codes used to define the comorbidities in the study cohort.

| Disease | ICD-10 Codes |
| --- | --- |
| Atrial Fibrillation | I48.0, I48.1, I48.2, I48.9 |
| Congestive heart failure | I50 |
| Chronic kidney disease | N18 |
| Chronic obstructive pulmonary disease | J41-J44 |
| Diabetes | E10-E11 |
| Hyperlipidemia | E78 |
| Hypertension | I10 |
| Ischemic heart disease | I20-I25 |
| Transient ischemic attack | G45 |

Subset of the codes after specified three digits were all included.

ICD-10 indicates International classification of disease 10^th^ Revision.

Supplementary Table 3. Billing codes used to define the predictors of stroke severity index.

| Predictors | Billing Codes |
| --- | --- |
| Airway suctioning | M0135-M0137 |
| Bacterial sensitivity test | C466 |
| General ward stay | AB100-AB697 |
| Intensive care unit stay | AJ001-AJ590 |
| Nasogastric intubation | Q2621 |
| Osmotherapy | 14800, 35440 |
| Urinary catheterization | M0050, M0060, M0065 |

Subset of the codes after specified four or five digits were all included.

Sub-billing codes beginning with these were also included where applicable.

Supplementary Table 4. Baseline demographic and clinical characteristics of excluded stroke patients from the Korean NHIS-National Sample Cohort.

|  | SAH  N=85 | ICH  N=182 | Other NIH  N=62 | CI  N=5,237 | Total*  N=5,566 |
| --- | --- | --- | --- | --- | --- |
| Male | 40 (47.1) | 103 (56.6) | 46 (74.2) | 2303 (44.0) | 2,492 (44.8) |
| Age, years (mean) | 56.9 | 58.4 | 61.1 | 66.6 | 66.1 |
| 18-44 | 15 (17.7) | 27 (14.8) | 7 (11.3) | 195 (3.7) | 244 (4.4) |
| 45-54 | 22 (25.9) | 44 (24.2) | 14 (22.6) | 588 (11.2) | 668 (12.0) |
| 55-64 | 25 (29.4) | 46 (25.3) | 9 (14.5) | 1201 (22.9) | 1281 (23.0) |
| 65-74 | 15 (17.7) | 39 (21.4) | 21 (33.9) | 1901 (36.3) | 1976 (35.5) |
| 75-84 | 7 (8.2) | 23 (12.6) | 11 (17.7) | 1216 (23.2) | 1257 (22.6) |
| 85+ | 1 (1.2) | 3 (1.7) | - | 136 (2.6) | 140 (2.5) |
| Residence Area |  |  |  |  |  |
| Seoul Metropolitan City | 24 (28.2) | 55 (30.2) | 13 (21.0) | 1023 (19.5) | 1115 (20.0) |
| Other Metropolitan Cities | 15 (17.7) | 40 (22.0) | 17 (27.4) | 1119 (21.4) | 1191 (21.4) |
| Non-metropolitan Area | 46 (54.1) | 87 (47.8) | 32 (51.6) | 3095 (59.1) | 3260 (58.6) |
| Insurance Type |  |  |  |  |  |
| Self-employed Insured | 24 (28.2) | 58 (31.9) | 24 (38.7) | 1661 (31.7) | 1767 (31.8) |
| Employed Insured | 50 (58.8) | 98 (53.9) | 33 (53.2) | 2933 (56.0) | 3114 (56.0) |
| Medical Aid Beneficiary | 11 (12.9) | 26 (14.3) | 5 (8.1) | 643 (12.3) | 685 (12.3) |
| Comorbidities |  |  |  |  |  |
| Atrial Fibrillation | - | 7 (3.9) | 4 (6.5) | 116 (2.2) | 127 (2.3) |
| CHF | - | 4 (2.2) | 2 (3.2) | 119 (2.3) | 125 (2.3) |
| CKD | - | 2 (1.1) | - | 63 (1.2) | 65(1.2) |
| COPD | 2 (2.4) | 9 (5.0) | 6 (9.7) | 286 (5.5) | 303 (5.4) |
| Diabetes | 8 (9.4) | 32 (17.6) | 14 (22.6) | 855 (16.3) | 909 (16.3) |
| Hyperlipidemia | 8 (9.4) | 22 (12.1) | 15 (24.2) | 891 (17.0) | 936 (16.8) |
| Hypertension | 18 (21.2) | 65 (35.7) | 34 (54.8) | 1392 (26.6) | 1509 (27.1) |
| IHD | 2 (2.4) | 10 (5.5) | 10 (16.1) | 541 (10.3) | 563 (10.1) |
| TIA | 1 (1.2) | 4 (2.2) | 4 (6.5) | 277 (5.3) | 286 (5.1) |

Values are numbers (percentage) unless stated otherwise.

*This number is patients who have visited an outpatient clinic more than three times with a stroke diagnosis code.

CHF: congestive heart failure; CI: cerebral infarction; CKD: chronic kidney disease; COPD: chronic obstructive pulmonary disease; ICH: intracerebral hemorrhage; IHD: ischemic heart disease (including myocardial infarction); NA: not applicable; NHIS: National Health Insurance Service; NIH: non-traumatic intracranial hemorrhage; SAH: subarachnoid hemorrhage; SSI: stroke severity index (ranging from 4.1 to 27.11; higher score indicates more severe status); TIA: transient ischemic attack.

Supplementary Table 5. Lifetime probability of developing different subtypes of stroke. CIR_18-84_ of different subtypes of stroke from 2006 to 2015.

|  |  |  | 2006-2007 | 2008-2009 | 2010-2011 | 2012-2013 | 2014-2015 |
| --- | --- | --- | --- | --- | --- | --- | --- |
| Male | SAH | Total Population | 0.87% | 0.99% | 0.90% | 0.79% | 0.79% |
|  |  | Prevalence 1 | 0.91% | 1.03% | 0.93% | 0.82% | 0.82% |
|  |  | Prevalence 2 | 0.94% | 1.05% | 0.95% | 0.84% | 0.84% |
|  | ICH | Total Population | 3.18% | 3.09% | 2.94% | 2.30% | 2.71% |
|  |  | Prevalence 1 | 3.35% | 3.24% | 3.08% | 2.42% | 2.85% |
|  |  | Prevalence 2 | 3.48% | 3.34% | 3.19% | 2.50% | 2.96% |
|  | Other NIH | Total Population | 1.41% | 1.81% | 1.43% | 0.77% | 0.60% |
|  |  | Prevalence 1 | 1.50% | 1.92% | 1.52% | 0.81% | 0.64% |
|  |  | Prevalence 2 | 1.58% | 2.02% | 1.60% | 0.84% | 0.66% |
|  | CI | Total Population | 19.49% | 19.03% | 16.89% | 16.95% | 14.96% |
|  |  | Prevalence 1 | 20.56% | 20.09% | 17.82% | 17.91% | 15.81% |
|  |  | Prevalence 2 | 21.41% | 20.94% | 18.56% | 18.69% | 16.49% |
| Female | SAH | Total Population | 1.44% | 1.46% | 1.36% | 1.33% | 1.57% |
|  |  | Prevalence 1 | 1.49% | 1.51% | 1.41% | 1.38% | 1.63% |
|  |  | Prevalence 2 | 1.53% | 1.55% | 1.45% | 1.42% | 1.68% |
|  | ICH | Total Population | 2.64% | 2.37% | 2.28% | 1.96% | 1.78% |
|  |  | Prevalence 1 | 2.77% | 2.49% | 2.39% | 2.06% | 1.87% |
|  |  | Prevalence 2 | 2.88% | 2.60% | 2.49% | 2.14% | 1.94% |
|  | Other NIH | Total Population | 0.36% | 0.60% | 0.42% | 0.44% | 0.43% |
|  |  | Prevalence 1 | 0.38% | 0.63% | 0.45% | 0.46% | 0.45% |
|  |  | Prevalence 2 | 0.40% | 0.66% | 0.47% | 0.48% | 0.47% |
|  | CI | Total Population | 16.17% | 14.39% | 12.29% | 11.58% | 9.87% |
|  |  | Prevalence 1 | 16.97% | 15.11% | 12.91% | 12.17% | 10.38% |
|  |  | Prevalence 2 | 17.69% | 15.76% | 13.46% | 12.72% | 10.85% |

Total population represents estimates calculated with age- and sex- corresponding population as a denominator, whereas prevalence 1 and 2 are estimates calculated with denominator adjusted with two different prevalence rates^1^.

CI: cerebral infarction; ICH: intracerebral hemorrhage; NIH: non-traumatic intracranial hemorrhage; SAH: subarachnoid hemorrhage.

Supplementary Table 6. Estimated mean survival for 10-year period from Kaplan-Meier (KM) method and semi-parametric extrapolation method.

| Stroke Type | Cohort size | 10-year survival based on  KM estimate (2006-2015) | Extrapolation from first 5 years of follow up (2006-2010) | Relative bias |
| --- | --- | --- | --- | --- |
| SAH | 567 | 87.21 (2.07) | 87.07 (1.91) | -0.15 % |
| ICH | 1,007 | 75.84 (1.63) | 74.94 (1.66) | -1.18 % |
| Other NIH | 257 | 83.18 (2.98) | 82.02 (3.53) | -1.39 % |
| CI | 5,090 | 85.91 (0.62) | 85.13 (0.66) | -0.90 % |
| All Types | 6,921 | 84.43 (0.55) | 83.64 (0.41) | -0.94 % |

Estimates are given as mean (SE) in months.

Relative bias = (estimate from extrapolation – KM estimate)/KM estimate.

CI: cerebral infarction; ICH: intracerebral hemorrhage; NIH: non-traumatic intracranial hemorrhage; SAH: subarachnoid hemorrhage; SE: standard error.

Supplementary Table 7. Sensitivity analysis for lifetime direct medical cost per case by different subtypes of stroke in Korea.

| Stroke Type | (ICD-10) | Expenditures covered by NHIS | | Copayment Expenses | |
| --- | --- | --- | --- | --- | --- |
|  |  | 5% Discount | 0% Discount | 5% Discount | 0% Discount |
| SAH | (I60) | 54,545 (3,342) | 68,848 (5,929) | 11,358 (1,032) | 14,524 (1,533) |
| ICH | (I61) | 66,274 (3,373) | 83,676 (6,073) | 13,883 (809) | 17,400 (1,221) |
| Other NIH | (I62) | 46,926 (3,373) | 56,746 (8,064) | 9,098 (886) | 11,028 (1,476) |
| CI | (I63) | 48,146 (1,583) | 55,762 (2,328) | 11,146 (320) | 12,788 (482) |

US$1 = 1131.2 KRW in 2015.

Values are mean (SE) in US$.

CI: cerebral infarction; ICD: international classification of diseases; ICH: intracerebral hemorrhage; LE: life expectancy; N: number; NHIS: National Health Insurance Service; NIH: non-traumatic intracranial hemorrhage; SAH: subarachnoid hemorrhage; SE: standard error.

# Reference

1. Kim, J. Y. *et al.* Stroke Statistics in Korea 2018: A Report from the Epidemiology Research Council of the Korean Stroke Society. (2019).
